# Editorial: Prokaryotic Communications, Volume II: From Macromolecular Interdomain to Intercellular Talks (Recognition) and Beyond

**DOI:** 10.3389/fmolb.2022.910673

**Published:** 2022-04-26

**Authors:** Chew Chieng Yeo, Manuel Espinosa, Tatiana Venkova

**Affiliations:** ^1^ Centre for Research in Infectious Diseases and Biotechnology (CeRIDB), Faculty of Medicine, Universiti Sultan Zainal Abidin, Kuala Terengganu, Malaysia; ^2^ Centro de Investigaciones Biológicas Margarita Salas, Consejo Superior de Investigaciones Científicas, Madrid, Spain; ^3^ Fox Chase Cancer Center, Philadelphia, PA, United States

**Keywords:** cell-cell communication, prokaryotic communications, intermolecular communication, intramolecular communication, molecular recognition

## Introduction

The first volume of this Research Topic on Prokaryotic Communications was launched in 2019 and managed to attract a total of 20 articles covering the wide scope of intracellular, cell-cell, cell-host and cell-environment communications, as summarized in our Editorial (Yeo et al., 2021). Having a follow-up or a sequel to such a relatively successful Research Topic was always going to be tough, particularly in a pandemic year marked by lockdowns which stymied experimental research in most parts of the world. Nevertheless, we managed to obtain six interesting articles which expands our knowledge on specific aspects of cell-cell, cell-host as well as intracellular communications. These are summarized below.

## Overview of Manuscripts in This Research Topic

Conjugative transfer, which is one of the main mechanisms for the spread of antimicrobial resistance genes, is mediated by type IV secretion systems (T4SS), a large membrane-spanning multiprotein complex that transfers single stranded DNA through the mating pair formation (MPF) complex into the recipient cell (Grohmann et al., 2018; Costa et al., 2021). The conjugative machinery of the broad host range Gram-positive plasmid pIP501 is encoded by a 15-gene *tra* operon (*traA* to *traO*) and in this Research Topic, Berger et al. focused on the small transmembrane protein TraB and demonstrated its essentiality in the conjugative transfer of pIP501. TraB, which has a secretion signal sequence, was also shown to interact with several MPF proteins and was postulated to function as a recruitment factor for other T4SS proteins which were unable to self-insert into the cytoplasmic membrane.

Multidrug efflux pumps have been strongly implicated in the antimicrobial resistance of many bacterial pathogens as they are capable of extruding antimicrobial compounds from the bacterial cytoplasm (Nishino et al., 2021). However, these efflux pumps have also been associated with various important physiological processes in bacteria, including in cell-cell communication (Henderson et al., 2021). Pasqua et al. reviews the role of efflux pumps of the Major Facilitator Superfamily (MFS) in host-pathogen communication and how they contribute to the virulence potential of several bacterial pathogens of concern including *Staphylococcus aureus*, *Klebsiella pneumoniae* and *Acinetobacter baumannii*. In another review paper, Sharma et al. presented the various types of biosurfactants known to be produced by bacteria and discussed their possible roles in a variety of cellular processes and cell-cell communication including quorum sensing, motility, virulence and biofilms.

Bacterial transcription is an essential intracellular communication process with the RNA polymerase holoenzyme (consisting of the RNA polymerase core enzyme plus the sigma factor) at its heart. Promoter recognition by RNA polymerase is reliant on the sigma factor with the housekeeping sigma factor being the one that is responsible for recognition of majority of promoters, particularly in exponentially growing bacteria (Lee et al., 2012). In the Gram-positive pathogen *Streptococcus pneumoniae*, housekeeping genes are recognized by the SigA sigma factor (also known as RpoD and σ^43^) but less is known regarding SigA as compared to the SigX (σ^X^) alternative sigma factor which functions to transcribe genes that mediate the development of natural competence for transformation (Håvarstein, 2010). Solano-Callado et al. investigated the *S. pneumoniae*-encoded SigA by purifying the native SigA protein and showed that it was able to not only recognize pneumococcal housekeeping promoters, but also promoters involved in the replication and mobilization of the *Streptococcus agalactiae* antibiotic-resistant plasmid pMV158.

The DnaA protein of *Escherichia coli* is the initiator protein that mediates chromosomal replication. Regulatory controls in the abundance and activity of DnaA are critical for precise cell cycle-coupled initiations such that replication initiation should only occur once per origin of replication (*oriC*) in each cell cycle, irrespective of growth conditions (Hansen and Atlung, 2018; Grimwade and Leonard, 2021). DnaA is a multi-domain protein with the AAA+ ATPase domain (Domain III) responsible for multimerization of DnaA on *oriC* with the ratio of ATP/ADP controlling the conformation and multimerization status of the protein. Charbon et al. showed that when cellular ATP/ADP ratio was lowered by carbon starvation, replication initiation was promptly arrested and DnaA is then degraded through a still unknown mechanism.

The *Bacillus subtilis*-encoded PcrA protein is part of the DNA helicase/translocase superfamily 1A (SF1A), is essential for DNA repair and repair-by-recombination processes, and thus its depletion is lethal in *B. subtilis* wild-type cells. In an earlier paper published in Volume I of this Research Topic (Moreno-del Alamo et al., 2020), showed that the cellular lethality due to inactivation or depletion of PcrA in *B. subtilis* could be suppressed by the inactivation of several genes leading the authors to suggest that PcrA functions in tandem with several recombination/repair proteins at stalled DNA polymerase/RNA polymerase complexes to facilitate replication beyond any conflict points that arise. Here in Volume II, Carrasco et al. showed that an iterative translocating PcrA monomer is capable of displacing its cognate RecA from single-stranded DNA (ssDNA) in presynaptic filaments to prevent it from catalyzing unscheduled recombination, thereby inhibiting DNA strand exchange (DSE) when it is not needed. The RecA mediators, SsbA and RecO, however balance such activity and if recombination is required, these mediators (SsbA, RecO, and RecR *in vivo*) will then stimulate rapid RecA filament reassembly. Nevertheless, how PcrA is recruited to RecA-bound ssDNA, and how SsbA and RecO could tilt the balance against the anti-recombinase activity of PcrA is still unknown.

## Conclusion and Perspectives

In this Research Topic on Prokaryotic Communications, we explored in depth certain areas of research that were presented in the preceding Volume I of this Topic. Taken as a whole, the articles that made up these two volumes have shown us that communication, be it intercellular or intracellular, along with molecular interactions lie at the heart of cellular processes. Although we have covered quite a distance in terms of the breadth and depth of our knowledge, we are still some ways to go to have a full understanding of the complexity of the milieu, or what we had described as the “intricate intra- and intermolecular dance” (Yeo et al., 2021) of the most abundant living organisms on planet Earth.
